# Lived experiences of pregnant and parenting adolescents in Africa: A scoping review

**DOI:** 10.1186/s12978-023-01654-4

**Published:** 2023-08-03

**Authors:** Anthony Idowu Ajayi, Sherine Athero, Winstoun Muga, Caroline W. Kabiru

**Affiliations:** Sexual, Reproductive, Maternal, New-Born, Child, and Adolescent Health (SRMNCAH) Unit, African Population and Health Research Center, Nairobi, Kenya

**Keywords:** Social exclusion, Challenges, Lived experiences, Pregnant and parenting adolescents, Adolescent mothers, Girls empowerment

## Abstract

**Background:**

Previous studies have not synthesized existing literature on the lived experiences of pregnant and parenting adolescents (aged 10–19) in Africa. Such evidence synthesis is needed to inform policies, programs, and future research to improve the well-being of the millions of pregnant or parenting adolescents in the region. Our study fills this gap by reviewing the literature on pregnant and parenting adolescents in Africa. We mapped existing research in terms of their substantive focus, and geographical distribution. We synthesized these studies based on thematic focus and identified gaps for future research.

**Methods:**

We used a three-step search strategy to find articles, theses, and technical reports reporting primary research published in English between January 2000 and June 2021 in PubMed, Jstor, AJOL, EBSCO Host, and Google Scholar. Three researchers screened all articles, including titles, abstracts, and full text, for eligibility. Relevant data were extracted using a template designed for the study. Overall, 116 studies met the inclusion criteria and were included in the study. Data were analyzed using descriptive and thematic analyses.

**Results:**

Research on pregnant and parenting adolescents is limited in volume and skewed to a few countries, with two-fifths of papers focusing on South Africa (41.4%). Most of the studies were African-led (81.9%), received no funding (60.3%), adopted qualitative designs (58.6%), and were published between 2016 and 2021 (48.3%). The studies highlighted how pregnancy initiates a cycle of social exclusion of girls with grave implications for their physical and mental health and social and economic well-being. Only 4.3% of the studies described an intervention. None of these studies employed a robust research design (e.g., randomized controlled trial) to assess the intervention’s effectiveness. Adolescent mothers' experiences (26.7%) and their education (36.2%) were the most studied topics, while repeat pregnancy received the least research attention.

**Conclusion:**

Research on issues affecting pregnant and parenting adolescents is still limited in scope and skewed geographically despite the large burden of adolescent childbearing in many African countries. While studies have documented how early pregnancy could result in girls' social and educational exclusion, few interventions to support pregnant and parenting adolescents exist. Further research to address these gaps is warranted.

**Supplementary Information:**

The online version contains supplementary material available at 10.1186/s12978-023-01654-4.

## Introduction

One group of young people that has received limited attention in sexual and reproductive health and rights research is pregnant and parenting adolescents (aged 10–19). This group of adolescents faces daunting challenges as they navigate parenthood, care for their babies, and improve their lives [1–3]. Many forfeit their future aspirations, including their educational goals and skills acquisition, with significant implications for their health and well-being, as well as that of their offspring [4–6].

Girls who become pregnant outside marriage often face stigma because of widespread socio-cultural and religious beliefs that sex should only occur in marriage [7]. As a result, some face an an hostile home environment or move away from home to reside with their partners [8]. Further, girls who become pregnant while in school are often forced to drop out of school [9]. This situation initiates a cycle of events culminating in their social exclusion. Social exclusion of pregnant and parenting adolescents can jeopardize the immediate and future health and well-being of young mothers. Patton et.al. argues that adolescence offers huge opportunities to alter negative and harmful trajectories that can jeopardize their future health. In their Lancet Commission, they demonstrate that investing in adolescent health, education and family would yield a triple dividend of benefits in the development of capabilities during adolescence, future adult-health trajectories, and secure welfare of the next generation [10].

Despite the existence of school reentry policies in most African countries that should facilitate reentry, available estimates show that a vast majority of pregnant and parenting adolescents are out of school even though they would like to return to school [11]. The few that manage to return to school describe the school environment as hostile, discriminatory, and inflexible [12]. Teachers unfairly target them, resulting in them dropping out or infrequently attending school [13].

Adolescent pregnancy and parenthood are also associated with child marriage, as these adolescents may be forced to move in with or get married to their partners [14, 15]. For some adolescent girls who become pregnant outside of wedlock, marriage serves as the only option to escape the associated stigma and social exclusion, enhance their status in the community or get financial support to care for their children [16]. However, child marriage can expose adolescent mothers to mental problems [17], school dropout [18], and intimate partner violence [19].

The social exclusion of pregnant and parenting adolescents has grave implications for gender equality. Because they rarely return to school [11], pregnant and parenting adolescents are unable to achieve their educational goals, which has consequences for their participation in the labor force [4]. Ultimately, this disenfranchises them and their children. Even though there are review studies on adolescent sexual and reproductive health, experience of adolescent mothers affected by HIV, and adolescent mental health in Africa [20, 21], there is limited attention to the lived experiences of pregnant and parenting adolescents. Given the unique challenges facing this cohort of adolescents, it is pertinent to review the literature to identify gaps in research on this population. Also, noting that a quarter of adolescent girls are either pregnant or parenting in the region, there is a need to target this population for interventions [22].

A review of existing studies will inform our knowledge of what interventions exist to improve their health and well-being as well as their socioeconomic and education empowerment and identify areas to prioritize for interventions. Our scoping review addresses this gap, drawing on the social exclusion framework [23], and aims to answer three questions: (1) What is the profile of research on the lived experiences of pregnant and parenting adolescents in terms of research designs, geographical distribution and substantive focus, including motherhood, and experiences in the community and schools? (2) How does early childbearing impact pregnant and parenting adolescents’ mental health? (3) What interventions are reaching adolescent mothers to improve their health and socioeconomic wellbeing?

## Methods

A scoping review is the appropriate design for the study given we aim to explore the breadth and extent of the literature, map and summarize the evidence, and inform future research, of the broader objective of this review.

### Search strategy

Guided by the Joanna Briggs Institute (JBI) methodological approach, we searched for peer-reviewed papers and grey literature published between January 2000 and June 2021 on pregnant and parenting adolescents. Grey literature was limited to theses. The search was limited to documents published in the English language and focusing on African countries. A three-step search strategy was used to ensure our search was comprehensive. First, we conducted a limited PubMed search to identify medical subject heading (MesH) terms for pregnant adolescents, adolescent mothers, and adolescent fathers. We analyzed the text words in the title, abstracts, and index terms in the articles from the initial search. We then created search terms for the study using the results of our analysis. In the second step, we searched PubMed, Jstor, AJOL, EBSCO Host, and Google scholar. After removing duplicates from the initial articles, we identified review studies found during our search. We reviewed the reference lists of these review articles and identified articles from the list that met our inclusion criteria. A detailed sample of PubMed search is provided in Additional file 1.

### Eligibility criteria

We included articles focusing on pregnant and parenting adolescents (married and unmarried) published in English between January 2000 and June 2021. As we aimed to identify gaps in research on pregnant and parenting adolescents with a specific focus on the challenges they face and interventions to address them, we only included articles that focused on parenting adolescents' well-being, including school reentry, livelihood, and repeat pregnancy, contraceptive use, mental health, motherhood challenges and care-seeking practices, and programs reaching pregnant and parenting adolescents. We excluded studies focusing on maternal health care services utilization, obstetric outcomes and adolescent pregnancy rate and risk factors to have a manageable number of articles and because previous systematic review studies have explored these topics [24–26] (Table 1).

**Table 1:** Table of eligibility criteria

Inclusion criteria	Exclusion criteria
Articles are written in English	Articles focusing on HIV prevalence and PMTCT
Articles published between 2000 and March 2021	Articles describing maternal health care services utilization
Articles focusing on an African country	Article focusing on breastfeeding
Articles focusing on pregnant and parenting adolescents (mothers and fathers)	Article describing obstetric outcomes
Articles identifying challenges faced and describing the lived experiences of adolescent parents	Articles focusing on adolescent pregnancy rate and risk factors
Articles focusing on repeat pregnancy and contraceptive use	Commentaries, books, editorials, conference abstracts
Articles focusing on coping strategies	Studies not focusing on Africa
Articles focusing on school reentry	Studies published before the year 2000

### Study selection

**Fig. 1:**
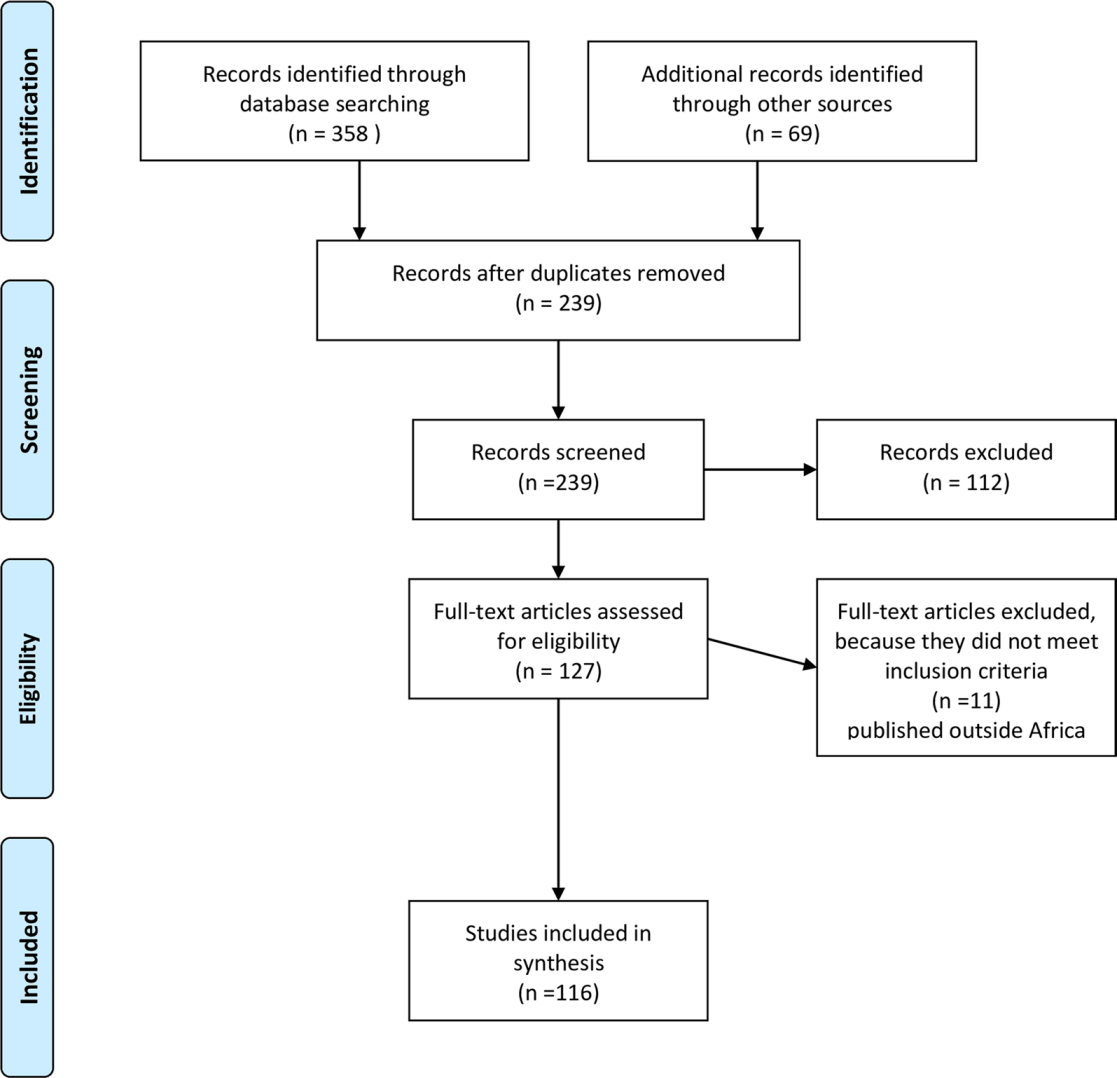
Overview of the articles selection process

Two reviewers independently screened the articles' titles and abstracts to assess their eligibility. Articles were included if they met the pre-specified inclusion criteria and if both reviewers agreed. When there was disagreement, a discussion was held with a third reviewer to resolve it. Figure 1 presents the PRISMA flow diagram demonstrating the process of article screening, inclusion, and exclusion. The initial search yielded a total of 427 articles, from which we removed 188 duplicates. After screening abstracts and titles, we excluded 112 articles that did not meet the eligibility criteria. We assessed the full text of a total of 127 articles and further removed 11 articles that did not meet the eligibility criteria, leaving 116 articles in our analysis.

### Data extraction and charting

We developed a data extraction template using Microsoft Excel. Three members of the research team completed the data extraction. Specifically, we extracted the country of affiliation of the first and last authors, country of study and sub-region, year of publication, journal of publication, study design, study objectives, key findings, and funder. We also classified the articles into common themes, including contraceptive use, mental health, lived experiences, education, social support, motherhood, care-seeking practices, and repeat pregnancy and HIV. One member of the research team reviewed samples of the extracted data for quality assurance.

### Evidence synthesis

We synthesized the data using descriptive statistics and content analysis. Descriptive analysis was used to describe the studies in terms of geographic distribution, year of publication, thematic focus, and research design. We summarized the key findings under the themes generated.

## Results

**Fig. 2:**
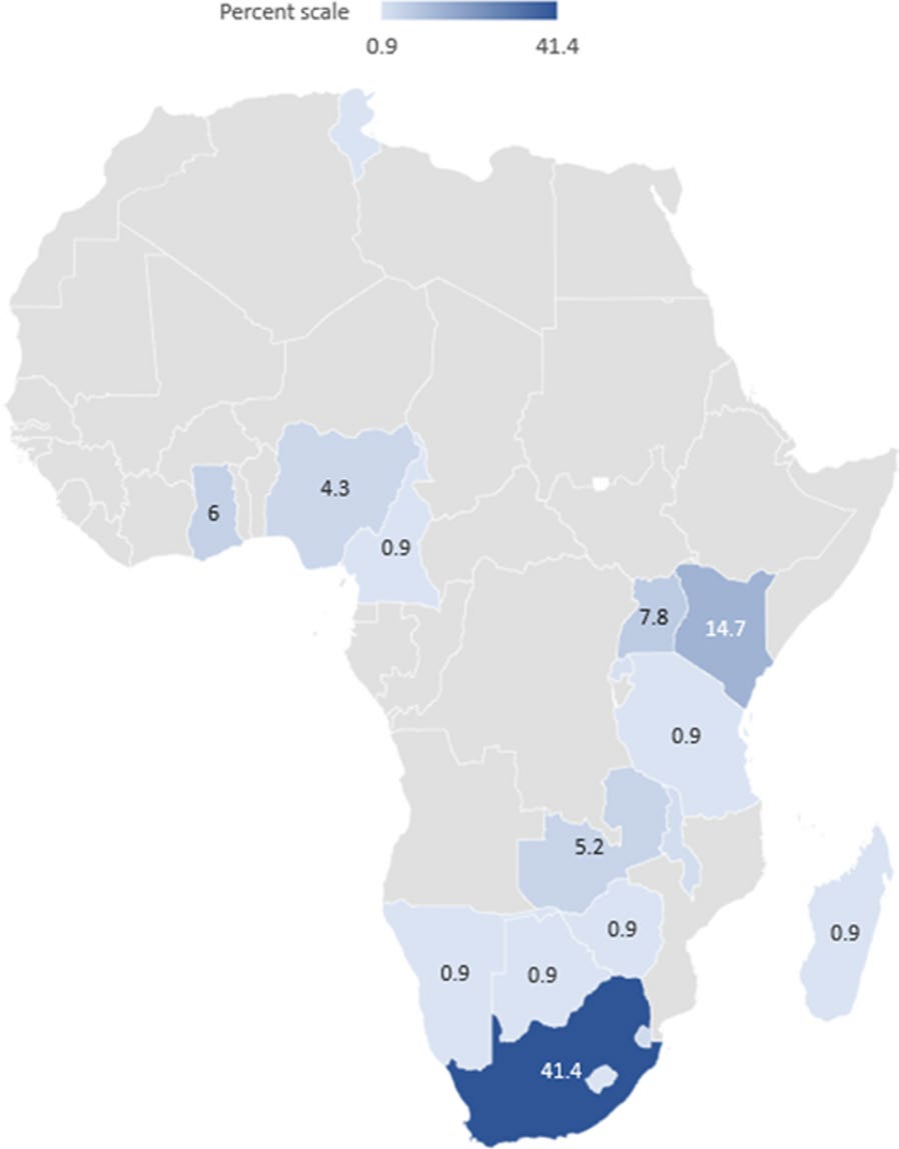
Geographical distribution of studies of adolescent mothers

Overall, 116 studies met the inclusion criteria and were included in the study. About half of the studies were published between 2016 and 2021 (48.3%). The studies were conducted in 17 African countries, and two-fifth of them focused on South Africa (41.4%) (Fig. 2). As shown in Table 2, most of the studies were African-led (81.9%). Only a few of the studies (4.3%) described an intervention, and none of these intervention studies employed a robust research design (e.g., randomized controlled trial design) to assess its effectiveness and impact. Qualitative methodology was the most commonly used study design (58.6%), enabling a deeper understanding of adolescent mothers' challenges. Adolescent mothers' experiences (26.7%) and their education (36.2%) were the most studied topics, while repeat pregnancy received the least research attention. Close to two in three studies did not receive any funding; 30.2% received external funding, while 9.5% had local funding. Organizations and agencies in the United States funded 37.1% of studies (n = 35) that received external funding. The United Kingdom (14.3%), Netherlands (14.3), Sweden (11.4%), and Canada (8.6%) governments were also prominent funders of research on pregnant and parenting adolescents in Africa.

**Table 2:** Characteristics of studies included

Variables	Frequency (n = 116)	Percent
Country/Region of affiliation of first authors		
USA	6	5.2
UK	1	0.9
Africa	95	81.9
Other European countries	9	7.8
Other countries like Brazil, Australia, New Zealand, and Canada	5	4.3
Country/Region of affiliation of last authors		
USA	8	6.9
UK	4	3.4
Africa	91	75.0
Other European countries	12	10.3
Other countries like Brazil, Australia, New Zealand, and Canada	5	4.3
Year of publication		
2000–2010	30	25.9
2011–2015	32	25.9
2016–2021	56	48.3
Sub-region		
East Africa	31	26.7
West Africa	14	12.1
Southern Africa	66	56.9
Sub-Saharan Africa	2	1.7
Global low and middle-income countries	3	2.6
Describe an intervention		
Yes	5	4.3
No	111	95.7
Study Design		
Qualitative study	68	58.6
Quantitative study	26	22.4
Review	10	8.6
Mixed methods	12	10.3
Publication type		
Journal	98	84.5
Thesis	14	12.1
Report	4	3.4
Thematic focus		
Family planning and contraceptive	8	6.9
Mental health	17	14.7
Motherhood and care-seeking practices	11	9.5
Lived experiences	31	26.7
Education	42	36.2
Social support	3	2.6
Repeat pregnancy	4	3.4
Funding information		
Not funded	70	60.3
Funded by institutions outside of Africa	35	30.2
Funded by an African Institution	11	9.5

### Partners, parents and community reactions to and support for pregnant and parenting adolescents

Partners, parents and community reactions to and support for pregnant and parenting adolescents were the areas that have received the most research attention [27–78]. However, eighteen of the 30 studies were conducted in South Africa. Most adolescent girls described their pregnancy as unintended [28, 29] and owing to transactional sex to meet their basic needs, sexual violence and exploitation, and lack of accurate information on methods of preventing pregnancy. A few adolescents wanted to become pregnant to command respect from people. Most were still in school when they became pregnant—many experienced denials of paternity [30, 31]. Boys were reported to deny paternity because they thought admitting it would jeopardize their educational and employment opportunities. As a result, adolescent mothers had limited support from the boys or their parents. Adolescent girls’ reactions to their pregnancy ranged from disappointment, anger, regret, and anxiety, for many, to a personal sense of satisfaction, happiness, and accomplishment, for a few [32].

Family reactions to adolescent pregnancy and motherhood were largely negative [30, 31, 33] and ranged from anger, disappointment, abandonment, rejection, and physical and emotional violence. In studies in Ghana and South Africa, parents and guardians of adolescent mothers were upset in the initial stages when they heard the news of the pregnancy, but they subsequently turned the initial emotion into forgiveness and acceptance. Lack of support from families, friends, and society was reported in Nigeria [33]. In Swaziland [32] and South Africa [34], adolescent pregnancy strained relationships with fathers, but mothers provided emotional and material support [32].

Adolescent mothers were noted to experience extreme hardship, educational disruption, stigma, stress, loneliness, guilt, and harsh treatment from family, schools, hospitals, and community members [35, 36]. They also faced financial constraints and food insecurity, prompting some to take up menial jobs. They faced unfavorable health [37] and school systems [38] emanating from discrimination by health workers, abuse and mockery from teachers, and stigma from peers [35]. This, in return, restricted them from effectively managing their schoolwork and parenting roles and resulted in delays in healthcare seeking and poor performance in school. Their pregnancy was seen as a major impediment to their education and career aspirations. Others were forced into early marriages and left feeling rejected. The negative treatment of pregnant and parenting adolescents was associated with skill gaps in handling parenting adolescent needs among key stakeholders, including parents, teachers, and service providers. Positive experiences included parenting adolescents’ views of their children as a source of meaning and the aspirations they had for their children [39]. Also, despite these challenges, adolescent mothers in South Africa were more likely to report parental support [28].

Only four studies focused on the experiences of adolescent fathers [40–43] and were conducted in South Africa. Peer influence, misconceptions about contraceptives, multiple partners, and low education attainment were associated with adolescent fatherhood. The studies found that adolescent fathers' own experience of absent fathers gave them a strong sense of responsibility towards their children and partners, but they faced financial constraints and were emotionally, psychologically, and socioeconomically overwhelmed by parental responsibilities. Adolescent fatherhood was related to stress and feelings of low confidence due to stigma related to becoming a father too early. Some had to work to support their children.

### School reentry policies and experiences of adolescent mothers in school

Forty-two studies focused on school reentry policies and adolescent mothers' experiences in school [5, 7, 11, 44–79]. These studies are mainly from South Africa (19 publications), Kenya (11 publications), Zambia (six publications), Ghana (two publications), Eswatini (one publication), Tanzania (one publication), Namibia (one publication), and Ghana (one publication). The review shows that most countries studied have school reentry policies in place while others are in the process of drafting or finalizing a policy [60]. In Kenya, there is a school reentry policy, but key stakeholders are unfamiliar with the provisions within the policy and are unable to fully implement policy [49]. Thus, 98% of adolescent mothers were reported to be out of school [56]. A lack of proper monitoring systems to ascertain conformity with the guidelines and limited circulation to headteachers and principals were noted [60]. In Zambia, pregnant girls were reported to drop out of school voluntarily or involuntarily as soon as the pregnancy is visible. On return to school, they experienced discrimination, mockery, abuse, humiliation, labeling, and isolation from teachers, peers, friends, classmates, and community members [7]. Their social exclusion resulted in low self-esteem, identity crises, poor academic performance, alcohol use, truancy, and running away from home [7]. Parents and adolescent mothers lacked information about the school reentry policy and guidelines resulting in the limited implementation of the policy [80]. Also, preference for boys’ education and poverty affected adolescent mothers' education with parents not willing or lacking resources to fund their education [57, 80].

While most adolescent mothers wanted to reenter school, they were constrained with child care responsibilities coupled with various contextual barriers, including financial burden, lack of emotional and social support, culture, lack of policy guidelines, fear of the school being ostracized by the community, fear of having mothers at school, and political factors, which impeded their full reintegration in school and impacted negatively on their school performance [11, 56, 60]. These contextual barriers suppressed the implementation of school reentry policies, especially in very conservative communities. Prevailing negative factors such as childcare responsibilities, poor economic background, and unsympathetic teachers and schoolmates made it difficult for adolescent mothers to reintegrate back into school [60]. Almost all adolescent mothers indicated that financial support for school fees and other expenses is critical for their reentry back to school. Only a few mentioned the need for childcare support. A study in Kenya found that school environment, teacher encouragement, school clubs, school sponsors, attitudes of other learners, the attitude of the school principal, teacher parenting program, curriculum, guidance and counseling services, opportunities to take part in activities and perform duties, motivational talks by resource persons, time for arrival and departure from school are factors facilitating the education of adolescent mothers [79].

In South Africa, however, many adolescent mothers returned to school. But, there were concerns that they return too early, as early as the first two months of postpartum [63]. Their return to school was fraught with challenges like limited support, social stigma, verbal abuse, and discrimination, resulting in many quitting schools or not succeeding with schooling [46, 48]. Adolescent mothers also struggled with balancing childcare with school demands [45]. But teachers were aware of their constitutional right to education and painstakingly protected this right [47].

### Mental health

Few studies have focused on the mental health of pregnant and parenting adolescents. Except for two studies, the rest were published between 2015 and 2021 [20, 81–96]. They concentrated on Zimbabwe [87], Nigeria [81, 85], South Africa [86, 92, 95], Kenya [83, 91, 93], Uganda [82, 88], Rwanda [94], and Lesotho [90]. One study looked at data from across sub-Saharan Africa [16], while one focused on global data[84]. Except for one intervention study, all these studies described the mental health challenges faced by pregnant, and parenting adolescents (including suicidal ideation, stress, anxiety, hearing voices, depression), the key stressors increasing their risk to mental distress, and challenges they experienced in accessing care.

These studies demonstrate that pregnant and parenting adolescents face high levels of depression, stress, and anxiety, heightened by their social exclusion, poverty, intimate partner violence, rejection by partners and parents after becoming pregnant, stigma from the community, chronic illnesses like HIV, and childhood vulnerabilities. The prevalence of depression ranged from 13% in Zimbabwe [87], 16% in South Africa [92], 48% in Rwanda [94], and 33% [93], and 53% [91] in Kenya. Common risk factors for depression included physical violence, verbal abuse, intimate partner violence [92], low family income, psychoactive substances [91], having experienced stressful life events, being diagnosed with HIV/AIDS, absence of social support, abandonment by a partner, absence of both parents during childhood [93], social insecurity, negative perception of teenage pregnancy, and bad relationships within families [87]. Protective factors included partner support [92]. Postpartum depression among adolescent mothers was associated with parental distress, weight/body shape disturbances, economic income, and parental-child dysfunctional interaction [94].

Negative service providers’ attitudes and stigma towards mental illness [85] and adolescent pregnancy, lack of confidentiality, and logistic and environmental challenges prevented the use of mental health services [86]. The lack of an all-inclusive approach to address adolescent parents’ multiple needs, including inadequate capacity and training for healthcare providers on handling their needs, was another challenge. Limited evidence exists on the effectiveness of psychosocial interventions on mental health disorders, prevention or treatment of common mental illnesses for adolescent mothers, particularly from low- or middle-income countries to inform effective intervention strategies for mental health illnesses.

### Motherhood challenges and care-seeking practices

A few studies focused on the challenges faced by adolescent mothers and care-seeking behaviors [97–107]. These studies were conducted in Uganda, South Africa, Swaziland, and Ghana with one study focusing on sub-Saharan Africa. The findings highlighted adolescent mothers' limited knowledge and skills about newborn/childcare practices. They often resorted to practices deemed harmful to their children [102], such as applying hot towels heated with hot stones to children's umbilical stump [102, 104]. Further, early motherhood was noted to strip adolescent girls of their agency and expose them to the stigma that compounds their barriers to accessing care during and after pregnancy. As a result, adolescent mothers may face more challenges during pregnancy and early motherhood than adult mothers.

### Contraception

Only seven studies focused on contraception among parenting adolescents [82, 108–113], four were published between 2001 and 2004, and two were published in 2020. These studies focused on contraceptive knowledge, attitude, perceptions, and use and were conducted in Cameroon, Nigeria, South Africa, Malawi, and Uganda. The studies highlighted low contraceptive uptake or the use of less effective methods like periodic abstinence, herbal concoctions, and vaginal douching [108]. However, in Uganda, Muyama et al. [82] reported a relatively high uptake of contraceptives among adolescent mothers, influenced by the desire to return to school [82]. The findings in terms of contraceptive knowledge were mixed with the study conducted in Nigeria, showing that contraceptive knowledge is poor. In contrast, the study in Cameroon found that most adolescent mothers had heard about contraceptives [111]. A study in South Africa reported several barriers to contraceptive use among parenting adolescents, including fear of side effects, partner rejection, providers' attitudes, and shortage of contraceptive supplies [112].

### Repeat pregnancy

Just three studies focused on repeat pregnancy among adolescent mothers, and all were conducted in South Africa [114–116]. The prevalence of repeat pregnancy in South Africa ranged from 17.6% to 19.9%. A history of spontaneous abortion, contraceptive use, a higher level of education, and emotional support were protective against repeat pregnancy. However, HIV-positive status, having more than one sexual partner and having a partner that is at least five years older were risk factors for repeat pregnancy. Adolescent mothers who received medical, psychosocial, educational, and family planning support experienced lower repeat pregnancy rates.

### Programs reaching pregnant and parenting adolescents

Five studies described programs targeting adolescent mothers [117–121]. Two of these five papers reported on one program—the Teenage Mothers project—implemented in Uganda [120, 121]. The program used an iterative, bottom-up, participatory approach to co-design an intervention to improve the psychological and social well-being of unmarried adolescent mothers. The program encompassed five intervention components: community awareness-raising, teenage mother support groups, formal education and income generation, counseling, and advocacy. The program was evaluated using qualitative research, and the findings suggest that it contributed to the teenage mothers' well-being and supportive social environment and community norms towards their future opportunities. Results also suggested that the program increased agency, improved coping with early motherhood and related stigma, continued education, and increased income generation. However, the program was not effective in changing community norms regarding out-of-wedlock sex and pregnancy [120].

Another study in Kenya used young mothers' clubs to increase adolescent mothers' knowledge of family planning and postpartum hemorrhage [118]. Young mothers participating in the program met weekly to share experiences and solutions to their challenges while receiving health education from health facility staff and community health workers.

Another intervention was implemented in Malawi to improve adolescent mothers' well-being and promote the healthy upbringing of their children. The program was informed by a literature review and consultation with key stakeholders. A safe space was created to share the daily challenges faced by adolescent mothers. Key stakeholders were brought to teach mothers about various topics like brain development, hygiene, and nutrition. Their children were provided with early childhood education and stimulation activities for up to two and a half years. Lastly, there was a community advocacy component to ensure the continued support of adolescent mothers.

As part of the fourth intervention, adolescent mothers in South Africa were introduced to kangaroo mother care to improve their childcare practices [119]. Kangaroo mother care is the practice of skin-to-skin contact between an infant and parent and has been found to improve growth and decrease the morbidity and mortality of low-birth-weight and premature infants [119]. Adolescent mothers in this intervention reported positive feelings about the kangaroo mother care. They also reported positive interactions with nurses, doctors, and other mothers and were pleased with the physical, emotional, social, and discharge support they received. However, they considered kangaroo mother care boring because they would just sit with their babies and do nothing.

## Discussion

Ours is the first study to our knowledge to synthesize existing literature on the experiences of pregnant and parenting adolescents in Africa. Research on pregnant and parenting adolescents is generally limited in volume and skewed to a few countries despite most countries recording a high prevalence of adolescent childbearing. The bulk of research on these adolescents is from two countries (South Africa and Kenya), underscoring the gaps in the geographical distribution of research on the issue in the region. These two countries are by far not the ones with the highest prevalence of adolescent childbearing in Africa. The significant research attention on the issues in these countries could make the issues facing pregnant and parenting adolescents more prominent to policymakers. Kenya, for example, formulated and released National Guidelines for School Reentry in Early Learning and Basic Education in 2020 [122]. Without the significant research attention on the issue in Kenya, this may have been impossible.

Despite the limited research attention on the experiences of pregnant and parenting adolescents, more recently, there appears to be increasing research attention given that about half of the studies were conducted between 2016 and 2021. Researchers' apparent growing interest in this population of adolescents bodes well for future research and investments in programs to improve their well-being. If the current interest in pregnant and parenting adolescents continues, there is a possibility for filling research gaps and addressing the wide geographical gaps in its distribution.

Unlike research on adolescent sexual and reproductive health in general [123], affiliated African authors led a vast majority of research on pregnant and parenting adolescents’ experiences and challenges. Adolescent sexual and reproductive has received significant research attention given it is one of the global development priority topics. Ending HIV, child marriage, female genital mutilation, early and unintended pregnancy, and increasing contraceptive uptake among adolescents are important global health priorities that have received significant research and program investments. However, issues affecting adolescent mothers have received limited focus, including funding and program investments. It is not surprising that most studies on pregnant and parenting adolescents did not receive any funding. The low representation of global north researchers in publications on these adolescents’ experiences and challenges may reflect the limited research funds available on these issues. Given that millions of girls in the region become pregnant every year, it is important that issues affecting them, particularly their education and skill empowerment, prevention of repeat pregnancy, mental health, and prevention of partner and non-partner violence, gain global attention. Empowering pregnant and parenting adolescents is key to realizing Sustainable Development Goal 5—Achieve gender equality and empower all women and girls. It is, therefore, imperative that global development partners and governments invest in research and programs to improve their health and well-being.

Our review demonstrates that pregnant and parenting adolescents, particularly girls, face several social, education, health, and motherhood challenges, including stigma, poor mental health, low contraceptive uptake, repeat pregnancy, lack of support, hostile school environment. Yet limited studies report on interventions to address these challenges. Such overwhelming neglect suggests that the suffering and social exclusion of this population of adolescents will continue, resulting in their disempowerment, poverty, and exacerbation of gender inequality. However, it is important to note that there is a range of experiences across the continent and challenges faced by adolescent parents varies hugely depending on the context [63]. For example, adolescent mothers are more likely to return to school in South Africa compared to Kenya, suggesting differential experiences of adolescent mothers in both settings [11, 63]. Also, some countries have formulated school reentry policies to address hostile school environments and facilitate adolescent mothers’ return to school.

While most studies focus on pregnant and parenting adolescents’ lived experiences and education, topics like contraceptive uptake, repeat pregnancy, intimate partner violence, mental health, and interventions to demonstrate what works in improving overall well-being and empowerment have received limited attention. Even though a few studies described an intervention, none used robust research designs to assess their effectiveness. Gaps exist in terms of understanding what works to empower adolescent mothers educationally and economically. Overall, evidence on scalable and cost-effective programmatic responses for adolescent mothers' education and economic empowerment is lacking in sub-Saharan Africa. Also lacking are studies documenting the complex nature of adolescent fatherhood and its impacts on their health and socioeconomic well-being. Overall, the studies were limited in scope and geographical distribution. There is a need for studies on lived experiences of pregnant and parenting adolescents in many African countries where no such studies exist. Future studies should document the positive experiences of pregnant and parenting adolescents and especially young fathers.

## Limitations

Our study is not without limitations. The articles reviewed are limited to those published in English. Excluding publications written in other languages may have potentially limited the number of studies reviewed. Our search was also limited to online sources and might have missed out on manuscripts not published online. We also did not assess the quality of the studies included. Lastly, since our search was completed in 2021, there is a need for future studies to update this review to keep pace with the evolving research on the topic.

## Conclusion

Our review shows that research on lived experiences of pregnant and parenting adolescents is limited in scope and geographical coverage. While studies have documented how early pregnancy could result in girls' social and educational exclusion, few interventions to support and empower pregnant and parentings adolescents exist. Further research is warranted on repeat pregnancy, contraceptive uptake, and exposure to violence among pregnant and parenting adolescents. Further, research on what works to empower these adolescents is needed.

### Supplementary Information

**Additional file 1.** Sample search terms.

## Data Availability

All data analysed are in the article.
