# Supplementary material for: Lived experiences of pregnant and parenting adolescents in Africa: A scoping review
Source: Reprod Health. 2023 Aug 3;20:113. doi: 10.1186/s12978-023-01654-4 (PMC10401816; doi:10.1186/s12978-023-01654-4)
Supplement: Supplementary file 1 — Additional file 1. Sample search terms. [file 12978_2023_1654_MOESM1_ESM.docx]

**Below is the example of our PUBMED search:**

**Search:** Search: ((((((("adolescent mothers") OR ("adolescent fathers")) OR ("young mothers")) OR ("teenage mothers")) OR ("early motherhood")) OR ("early fatherhood")) OR ("teenage fathers")) AND (((((((((((Africa) OR (sub-Saharan Africa)) OR (west Africa)) OR (east Africa)) OR (southern Africa)) OR (Nigeria)) OR (Ghana)) OR (kenya)) OR (malawi)) OR (central Africa)) OR (Angola OR Benin OR Botswana OR "Burkina Faso" OR Burundi OR "Cape Verde" OR Cameroon "Central African Republic" OR Chad OR Comoros OR Congo OR "Democratic Republic of the Congo" OR "Cote d'Ivoire" OR "ivory coast" OR Djibouti OR "Equatorial Guinea" OR Eritrea OR Eswatini OR Swaziland OR Ethiopia OR Gabon OR Gambia OR Ghana OR Guinea OR Guinea-Bissau OR Kenya OR Lesotho OR Liberia OR Madagascar OR Malawi OR Mali OR Mauritania OR Mauritius OR Mozambique OR Namibia OR Niger OR Nigeria OR Rwanda OR "Sao Tome and Principe" OR Senegal OR Seychelles OR "Sierra Leone" OR Somalia OR "South Africa" OR "South Sudan" OR Sudan OR Tanzania OR Togo OR Uganda OR Zambia OR Zimbabwe)) Filters: from 2000 - 2021
